# Role of Placental VDR Expression and Function in Common Late Pregnancy Disorders

**DOI:** 10.3390/ijms18112340

**Published:** 2017-11-06

**Authors:** Julia Knabl, Aurelia Vattai, Yao Ye, Julia Jueckstock, Stefan Hutter, Franz Kainer, Sven Mahner, Udo Jeschke

**Affiliations:** 1 Department of Obstetrics and Gynecology, University Hospital, Ludwig-Maximilians Universität München, 80337 Munich, Germany; Julia.knabl@gmx.de (J.K.); Aurelia.vattai@med.uni-muenchen.de (A.V.); yao.ye@med.uni-muenchen.de (Y.Y.); julia.jueckstock@med.uni-muenchen.de (J.J.); Stefan.hutter@med.uni-muenchen.de (S.H.); sven.mahner@med.uni-muenchen.de (S.M.); 2 Department of Obstetrics and Gynecology, Klinik Hallerwiese, 90419 Nürnberg, Germany; franz.kainer@diakonieneuendettelsau.de

**Keywords:** vitamin D, VDR, preterm birth, GDM, preeclampsia, fetal growth restriction

## Abstract

Vitamin D, besides its classical role in bone metabolism, plays a distinct role in multiple pathways of the feto-maternal unit. Calcitriol is the major active ligand of the nuclear vitamin D receptor (VDR). The vitamin D receptor (VDR) is expressed in different uteroplacental parts and exerts a variety of functions in physiologic pregnancy. It regulates decidualisation and implantation, influences hormone secretion and placental immune modulations. This review highlights the role of the vitamin D receptor in physiologic and disturbed pregnancy, as preeclampsia, fetal growth restriction, gestational diabetes and preterm birth. We discuss the existing literature regarding common VDR polymorphisms in these pregnancy disorders.

## 1. Introduction

The vitamin D endocrine system was formerly known as a key player in calcium and phosphate homoeostasis and in regulation of bone remodelling [1]. Hence, nowadays it is common knowledge, that the vitamin D endocrine system takes part in many non-classical pathways. Vitamin D influences maternal and fetal cell differentiation and cell growth, immune regulation, insulin secretion and anti-proliferative processes [2,3,4,5]. Therefore, this ligand is of special interest as it is involved in many diseases like autoimmune disorders, and type 2 diabetes mellitus (T2DM) [6,7].

The name vitamin is misleading, as in humans, only a small amount of vitamin D is obtained through dietary intake, while vitamin D is largely generated in the skin with exposure via photochemical conversion of 7-dehydrocholesterol to pre-vitamin D3, and the latter is sequentially metabolized in the liver and kidneys [8]. Extrarenal 1,25(OH)_2_D_3_ is metabolized by the colon, pancreas, immune system, endothelial cells and the placenta [9].

### 1.1. Vitamin D Receptor (VDR) Signaling

1α,25-(OH)_2_D (calcitriol) is the major active ligand of the Vitamin D receptor (VDR). VDR is part of the nuclear steroid hormone receptor family; and recruits another receptor of this group, for example, retinoid X receptor α to form a heterodimer. This complex works as a transcriptional activator of numerous genes, via VDR response elements of the targeted genes [10]. However, vitamin D can also exert rapid non-genomic effects, probably via VDR located within the plasma membrane [11,12].This rapid pathway works via specific enzymes as protein kinase C and mitogen-activated protein kinase [13]. They regulate cell proliferation and cell differentiation, invasive processes and apoptosis. 

### 1.2. VDR Gene and Polymorphisms

VDR is encoded by a capacious gene (>100 kb), it lies on the chromosome 12q12-14 [14]. The *VDR* gene includes two promoter regions, eight coding exons (namely, 2–9), and six untranslated exons (1A–1F). In the *VDR* gene, single nucleotide polymorphisms (SNPs) occur quite frequently [15]. Every genetic variant is called a polymorphism if it appears in at least 1% of the population. Recently, multiple loci in VDR binding proteins and the modulating enzymes *CYP2R1*, *CYP24A1*, *CYP27B1*, and *CYP27A1* genes have been linked to vitamin D levels [16,17].

**Figure 1:**
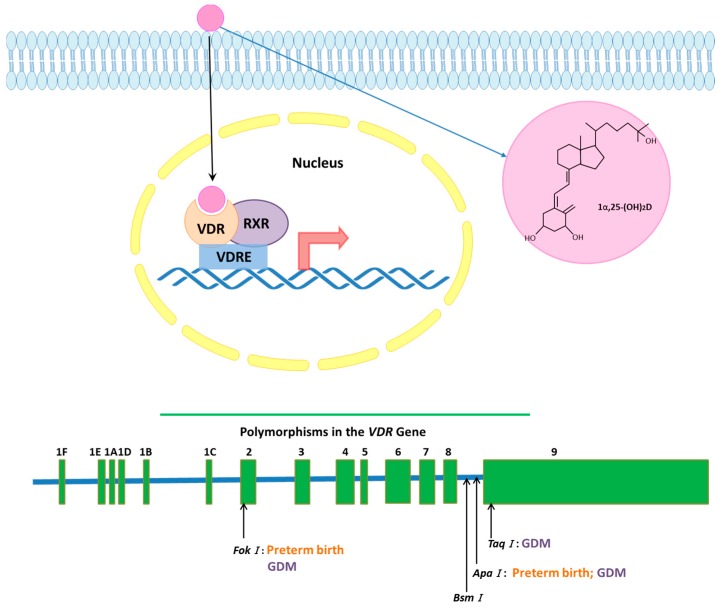
Vitamin D receptor expression and polymorphism: Vitamin D receptor (VDR) is a part of the nuclear steroid family and is also expressed in the plasma membrane. The major active ligand of VDR is 1α,25-(OH)_2_D (calcitriol). In nucleus, VDR recruits retinoid X receptor (RXR) to form a heterodimer, which binds to vitamin D response element (VDRE) and modulates the transcription of numerous genes. *VDR* gene is located on the chromosome 12q12-14, which consists of eight protein exons (namely, **2**–**9**) and six untranslated exons (**1A**–**1F**). *VDR* has four well-characterized di-allelic polymorphisms: *BsmI* and *ApaI* on the last intron, *FokI* and *TaqI* on the coding sequence. Among these polymorphisms *ApaI*, *FokI* and *TaqI* are linked to gestational diabetes mellitus (GDM), *ApaI* and *FokI* are linked to preterm birth.

Since the discovery of the *VDR* gene, common SNPs have been specified in the gene [15]. *VDR* gene has four precise described di-allelic polymorphisms: *BsmI* (A > G, rs1544410) and *ApaI* (A > C, rs7975232) on the last intron, and *FokI* (C > T, rs10735810) and *TaqI* (T > C, rs731236) polymorphisms lie on the coding exons [18,19]. Several studies investigated these SNPs and associated them with a couple of diseases [15,20,21] Therefore, *VDR* allelic variants and different expressions of the receptor play a key role in a number of health concerns, as e.g., breast cancer or auto-immune diseases [22]. Hence, the role of *VDR* and its allelic variants in pregnancy is not yet clarified [23]. A graphical view of VDR activation and its polymorphisms is presented in Figure 1.

The aim of this review was to summarize the existing knowledge about the role of VDR expression, function and polymorphisms in common pregnancy disorders.

## 2. VDR Expression in Physiologic Pregnancy

Trophoblasts are a major source of 1,25[OH]_2_D_3_ and during pregnancy it is elevated two folds [5]. It regulates decidualisation, implantation, lactogen expression. Furthermore, Vitamin D influences human chorionic gonadotropin (HCG), progesterone and estrogen secretion, calcium uptake into the placenta and placental immune modulations [24,25,26,27].

Placenta is also a major site for conversion of 25(OH)D_3_ to 1,25(OH)_2_D_3_ via CYP27B1 [28]. Placental calcitriol synthesis begins early in gestation, as placental expression of *CYP27B1* mRNA is already high in the first trimester [28]. Increased levels of Calcitriol suppresses *CYP27B1* transcription in trophoblasts [29,30], in contrary *CYP24A1* expression is upregulated. This effect works via ligand-bound VDR [29].

VDR expression was detected in villous trophoblast and decidua [31,32] and in smooth muscle cells of the placental vessels (VSMC) [2]. Nuclei of stromal cells in fetal villi and in the nuclei of fetal endothelial cells express also VDR [32]. VDR is known to regulate immune responses: Calcitriol stimulates the synthesis of cathelicidin (CAMP), an antimicrobial peptide, in trophoblasts, decidual cells and placental macrophages [30]. 1,25(OH)_2_D_3_ decreases synthesis of cytokines including tumor necrosis factor, granulocyte-macrophage colony stimulating factor, and interleukin-6 in decidua [33], these results indicate that the vitamin D system may play an important role in controlling placental responses to infection. For example, placental synthesis of 1,25(OH)_2_D_3_ is induced by lipopolysaccharides (LPS), a Toll-like receptor 4 ligand (TLR4) by upregulation of *CYP27B1* in mouse placenta [34].

Therefore, placental vitamin D system, including VDR, its ligands and the metabolizing enzymes like CYP27B1 plays a key role, possibly in combination with other factors like cytokines to maintain innate immunity and favours implantation [25,35].

## 3. VDR and Preeclampsia

Preeclampsia is defined by maternal hypertension, proteinuria and endothelial dysfunction, this syndrome affects up to 8% of pregnancies. It causes significant maternal and perinatal morbidity and mortality [36]. This accounts mostly for the severe, early onset form of preeclampsia, which has important pathophysiological overlaps with fetal growth restriction, and usually comes along with the latter. Placental insufficiency is a key characteristic of severe, early onset preeclampsia, and in addition of pregnancies with fetal growth restriction (FGR). Decreased trophoblast invasion, impaired placentation, disturbed remodeling of uterine arterioles [37], reduced cytotrophoblast proliferation and increased apoptosis [38] are specific observations in placental insufficiency. 

Vitamin D deficiency is connected to placental insufficiencies like preeclampsia and fetal growth restriction. Several observational studies found significant associations between Vitamin D levels and an elevated risk of preeclampsia or gestational hypertension diseases [39,40,41,42], but overall results are conflicting: Three cohort studies and three case–control studies weren’t able to identify a link between plasma 25(OH)D concentration and preeclampsia. Observations linked maternal Vitamin D and early onset and severe preeclampsia, but there was no association between maternal Vitamin D and late onset preeclampsia or overall preeclampsia risk [42,43]. However, the results are difficult to compare because of heterogeneity of populations, geographic location and lack of technical standardization in measurements. Other confounding factors, which influence vitamin D levels, are ethnicity, dietary habits, lifestyle and gestational age at sampling.

### 3.1. VDR Expression Changes in Preeclampsia

As to the specific placental metabolism, preeclampsia has recently been associated with decreased activation, increased catabolism, and impaired placental uptake of 25(OH)D_3_ [44]. In preeclampsia, which has significant pathophysiological overlaps with FGR, VDR expression is decreased [45,46,47]. Furthermore, placentas of preeclamptic women have reduced CYP27B1 enzyme activity in comparison to regular pregnancies [13]. One hypothesis connecting low vitamin D to the pathophysiology of preeclampsia is that vitamin D deficiency causes imbalances between Th1 to Th2 cytokines, while higher Th1 cytokine impairs embryo implantation [48]. Disturbed extravillous trophoblast (EVT) invasion of decidua and maternal spiral arteries is a key feature of preeclampsia and gestational hypertension. Recent data showed that calcitriol and calcidiol significantly increased EVT invasion in vitro [49]. Furthermore, in vitamin D deficiency is endothelial function of placental vessels disturbed and VDR expression on placental endothelium disturbed [23,50]. As genetic and epigenetic factors regulate protein expression, previous studies have reported that the placental Vitamin D concentration is influenced by epigenetic DNA methylation especially *CYP24AI* [51,52]. Therefore, hypermethylation of *VDR* gene may cause the downregulation of placental VDR in FGR-or preeclampsia.

### 3.2. Role of VDR Polymorphisms in Preeclampsia

There is a strong association between common *VDR* polymorphisms, as e.g., *BsmI* and *FokI*, and hypertension risk outside pregnancy [53,54,55]. In cell cultures, VDR-dependent signaling directly suppressed renin gene transcription [56]. *FokI* polymorphism of *VDR* influences plasma renin activity [57] and seems to be associated with a decreased risk of hypertension. Although there is also some evidence for a genetic contribution to hypertensive disorders of pregnancy [58,59,60], a recent case-control study found the three common *VDR* SNPs (*FokI*, *ApaI* and *BsmI*) equally distributed in gestation hypertension groups compared with healthy pregnancy cohort. In this study, neither these *VDR* genetic polymorphisms nor *VDR* haplotypes predisposed to preeclampsia or gestational hypertension [59].The authors concluded that other *VDR* polymorphisms might affect the risk of getting preeclampsia or gestational hypertension, as the *VDR* gene is very large (over 100,000 base pairs) [56,61].

## 4. VDR and Fetal Growth Restriction

Fetal growth restriction (FGR) is a major health concern as it applies up to 5% of all pregnancies worldwide and is an important cause of perinatal mortality and morbidity [62]. Defining criteria include birth weight of less than the 10th centile for gestation and other signs of danger for the fetus, as e.g., low amniotic fluid s or asymmetric fetal growth [63]. A growth retarded fetus misses its genetically predetermined size for gestational age [64], this is the important difference too small for gestational age infants (SGA). FGR causes perinatal complications, for example an increased rate of stillbirth or prematurity. These children are at higher risk for several diseases, including cardiovascular disease and diabetes [65,66], asthma [67], or neurological sequelae as intellectual developmental delay [68], schizophrenia [69], depression [70]. 

FGR has multiple underlying reasons, as e.g., fetal chromosomal abnormalities, maternal nicotine abuse and malnutrition. However ~70% of the cases are called idiopathic FGR as there is no evident reason to identify [71]. Characteristic signs of idiopathic FGR include is impaired function of uteroplacental vessels, including disturbed placental development and reduced flow from uterine vessels to the placenta [72]. The molecular pathophysiology is still not completely understood.

The possible mechanisms, which link vitamin D to fetal growth, are calcium metabolism, bone growth or altered placental function [73]. Vitamin D regulates via the VDR pathway human chorionic gonadotropin expression and secretion in human synctiotrophoblasts [2] and increases placental sex steroid production [24]. As it is explained in detail later in the manuscript, Vitamin D is also important in glucose/insulin homeostasis and for transplacental transport and fetal usage of glucose [74]. 

A systematic review linked maternal 25(OH)D and fetal growth by ultrasound [75] and several observational studies found associations between vitamin d levels and fetal growth [76,77,78]. Nevertheless, the results of the observational studies are conflicting: A multiethnic cohort study of pregnant women with vitamin D deficiency couldn’t identify a link between maternal vitamin D levels and any of the neonatal anthropometric measures [79].

FGR placentae show significant different placental morphology such as reduced villous tree elaboration and diminished surface area [38]. In addition, trophoblast invasion and cytotrophoblast proliferation [38] is reduced, cytotrophoblast apoptosis [80] is increased. Additionally, the fusion of the villous trophoblast forming the multi-nucleated syncytiotrophoblast is impaired in placenta of FGR [37,81].

### 4.1. VDR Expression and Signaling in Fetal Growth Restriction

VDR regulates cell proliferation and differentiation [82]. As mentioned above, VDR varies during pregnancy [31]. Placental VDR expression is decreased in human FGR and causes trophoblast dysfunction [83]. Therefore, decreased VDR expression may impair the actions and limits the beneficial effects of vitamin D in the regulation of feto-placental growth. 

In in vitro studies, VDR plays critical roles in the maintenance of proliferation, migration, differentiation and apoptosis of the trophoblasts. This may be a link between the pathology of idiopathic FGR-affected pregnancies and the reduced VDR expression in such cases [23]. Placentae of FGR patients show impaired villous trophoblast fusion forming the syncytiotrophoblast [37,81,84]. VDR is a critical regulator of placental hormone secretion e.g., placental lactogen and β-hCG in BeWo cells. BeWo cells work well as in vitro model for the syncytiotrophoblasts [27,85,86]. VDR regulates BeWo cell differentiation via the influence on β-hCG expression. Furthermore, VDR inactivation affects syncytium formation in vitro and VDR inactivation promotes apoptosis, using *TP53* mRNA as a marker of apoptosis [83]. Trophoblast syncytialisation is connected to decreased CYP27B1 in vitro [87]. VDR plays a role in protection against extrinsic apoptosis in placental insufficiency and maintains proper trophoblast function under adverse conditions like FGR [23]. As mentioned above, genetic and epigenetic regulations influence vitamin D effects on feto-placental development [88] and methylation changes in the *VDR* gene may reduce VDR expression in FGR [51,52].

### 4.2. VDR Polymorphisms in Fetal Growth Restriction

In addition, decreased expression of VDR in FGR may be due to SNPs of *VDR*, as polymorphisms influence expression and function of VDR [22,89]. *VDR* polymorphisms have been shown to modify offspring size; this was shown for the *FokI* genotype, but not for *ApaI*, *BsmI*, *TaqI* genotype. The latter have a high linkage disequilibrium in Caucasian population [90]. The *ApaI* polymorphism (rs7975232) is a non coding polymorphism of the *VDR* gene. Along with *TaqI* polymorphism (rs731236) this SNPs are associated with variation in mRNA stability [18]. As this influences the amount of protein, they might be able to change vitamin D levels and calcium homeostasis. However, the effect of a distinct maternal polymorphism on birthweight seems to differ across racial groups. In a prospective cohort study in North Carolina, a strong correlation between *ApaI* (rs7975232) and birthweight was identified in black mothers, but there was no association between any *VDR* SNP and birthweight for white mothers [91]. Common polymorphisms of *VDR*, which are associated with pregnancy complications, are listed in Table 1.

## 5. VDR and Diabetes in Pregnancy

Gestational diabetes mellitus is a state of pronounced peripheral insulin resistance, which results in glucose intolerance in the second half of pregnancy. This concerns 3% to 8% of all pregnancies depending on geographical location [92,93,94]. On one hand, GDM increases the risk of adverse pregnancy outcomes, as e.g., intrauterine fetal demise, fetal macrosomia, birth trauma and preeclampsia [95]. On the other hand, GDM influences health issues in later life of both mothers and their offspring, as overweight, type 2 diabetes mellitus (T2DM) and metabolic syndrome [96,97]. Vitamin D deficiency has been linked with insulin resistance in pregnancy [98] and with an increased risk of GDM [8]. Several studies have reported lower Vitamin D levels in women with GDM [8,99,100] or in the first half of pregnancy among women who later developed GDM. However, these results are controversial [101,102], other studies didn’t see an association between Vitamin D levels and GDM [102,103]. The influence of individual, lifestyle and geographical factors on Vitamin D status is complex. Skin pigmentation, sun exposure, adiposity and diet are known to influence vitamin D. In addition, physical activity increases sun exposure and decreases body weight, as well as potentially the risk of GDM. In a large birth cohort study women with vitamin D deficiency had higher odds of GDM [104], but that this association diminished after adjustment for different confounders. Overall, systematic reviews and meta-analyses found a modest raise for the risk of GDM [73,105]. The outcome of these analyses are restricted by cohorts from different regions, the varying laboratory standards and timing of measurement of serum 25(OH)D level. The quantity of the effect of some influencing factors like ethnicity and adiposity are still unclear [106]. Until now, the exact mechanisms underlying the association of vitamin D and insulin resistance are not fully understood: In general, Vitamin D regulates about 3% of the human genome, especially genes that influence glucose and lipid metabolism [107,108]. The following models link the pathogenesis of GDM with low levels of vitamin D [109,110,111,112]: Several studies demonstrated specific receptors for vitamin D in pancreatic β cells [113] and a role for vitamin D in the secretion of insulin [114,115]. It also has been reported that vitamin D deficiency is associated with insulin resistance and T2DM [116,117].

Vitamin D regulates the balance between extra- and intracellular calcium. Calcium is an essential co-factor for insulin-mediated intracellular functions in insulin-depending tissues such as skeletal muscle and adipose tissue [118,119,120]. It is responsible for adequate insulin-mediated functions [121]. Changes in intracellular calcium contribute to peripheral insulin resistance as Calcium has to be in a very distinct range [121]. Insulin receptor phosphorylation is a calcium depending function as well [122]. Changes in Calcium concentration influences insulin signal transduction [120,123] and leads to decreased glucose transporter GLUT-4 activity [123,124].The promoter region of the human insulin gene contains Vitamin D response element (VDRE) [123,125] and so transcription of the human insulin gene is upregulated by 1,25(OH)D_2_ [126].

VDR influences glucose homeostasis via the insulin-like growth factor system. Vitamin D has an impact on the immune system [127] as VDRE is part of the promoter region of certain cytokines and has impact on cytokine generation and action [128,129,130] as for example NF-kB [130,131]. This is an important regulator of genes encoding pro-inflammatory cytokines implicated in insulin resistance [132] or calbindin [133,134], a cytosolic calcium-binding protein found in many tissues including pancreatic beta cells [134]. GDM is a proinflammatory state like preexisting diabetes [135]. Deficiency in immune modulations e.g., via itamin D deficiency causes an severe inflammatory response, which is an essential part of insulin resistance [136].

### 5.1. VDR Expression in Gestational Diabetes Mellitus

As to placental VDR expression changes in GDM, our recent work showed increased levels of VDR in extravillous trophoblasts and fetoplacental endothelium associated with maternal GDM [93]. Vitamin D protects endothelial tissue against renovascular dysfunction [137]. As Vitamin D induces NO production in endothelial cells, and endothelial dysfunction in GDM is accompanied by increased NO production, this link seems probable for GDM. Possibly, upregulation of VDR in the fetoplacental endothelium is a result of low vitamin D levels even in the fetus [93].

EVT displayed the strongest VDR upregulation of all placental parts [93]. EVT, which forms the fetomaternal interface, prevents allo-recognition and attacking of fetal cells by maternal natural killer cells, cytotoxic T cells, and macrophages [138]. As shown above vitamin D supports the immune system in both maternal and fetal compartments [33,34,139] and it is a key regulator of placental inflammation [34]. GDM is an pro-infammatory state and VDR upregulation seems to compensate Vitamin D deficiency. A different study from Cho and co-workers [140] found no differences in VDR expression in GDM placenta, but they did not separate villous and extravillous trophoblasts. They found increased expression and production of CYP24A1 from patients with GDM compared with normal placental tissues and that serum vitamin D level was correlated negatively with the expression of CYP24A1 in placenta. They concluded that increased placental expression and production of CYP24A1 may be responsible for the low level of vitamin D that is observed in GDM [140].

### 5.2. VDR Polymorphisms and Gestational Diabetes Mellitus

Available data suggest that GDM has genetic endowments in combination with environmental impacts [141]. Mothers with GDM often have a family history of diabetes and GDM recurs in at least 30% of women with a history of GDM [142,143], even in different populations [144,145]. *VDR* polymorphisms have been linked to a higher risk for type 1 diabetes mellitus (T1DM) [146,147] and type 2 diabetes mellitus (T2DM) [148]. As GDM and T2DM have a similar pathophysiology [149], polymorphisms in genes predisposing for diabetes mellitus Type 2 are closely related to GDM susceptibility [150]. Some *VDR* polymorphisms are strongly influencing the susceptibility to diabetes in general [148,151]: *Bsm*IBB, *Bsm*IBb, and *Taq*Itt polymorphisms are linked to susceptibility of T1DM [152]. However, the connection between *Bsm*I SNP and T2DM is weak. In a Chinese cohort, F*ok*I polymorphism was significantly linked to T2DM risk, but not in Caucasians [153]. 

For the effect of *VDR* polymorphisms in GDM, only a few studies are available. A link between *VDR Apa*I, *Taq*I, and *Fok*I SNPs and risk for GDM was suggested in Iranian, Chinese and Saudi Arabian population [144,145]. But results with contrasting findings are available, as El-Beshbishy et al. couldn’t link *VDR BsmI* and *FokI* polymorphism and GDM in a Saudi population [154]. In Chinese female genetic variants of Vitamin D binding protein and the heterodimers RXRα and γ had been identified as susceptibility markers for GDM [155]. See also Table 1 for common polymorphisms of VDR, which are associated with pregnancy complications.

To conclude, further studies are warranted with sufficient statistical power and with different ethnic background in order to confirm the potential of genetic biomarkers for prediction of GDM in different populations.

## 6. VDR and Preterm Birth

Preterm birth (PTB) is one of the leading problems in modern obstetrics. Deaths among premature infants account for 70% of all perinatal mortalities. The surviving ones are at risk for chronic lung disease, hearing, visual and cognitive impairments [156]. Hence, the pathophysiology of preterm birth is still unknown and heterogeneous, obviously intrauterine infection is a frequent and important mechanism causing early delivery [157]. Pro-inflammatory cytokines and genes probably trigger delivery at term [158]. Therefore, early excessive cytokine productions because of infection can lead to preterm labor.

Even though Vitamin D modulates immune responses and plays an important role in defending infections [127,159], findings from randomized [160] and epidemiologic studies of Vitamin D and preterm birth are controversial [76,161,162,163,164]. However, a very recent meta-analysis of 18 studies of either observational or interventional design, found sufficient evidence linking Vitamin D insufficiency with PTB. Serum 25(OH)D levels <75 nmol/l was associated with 13% and 83% increased risk of PTB measured at <35–37 weeks and <32–34 weeks, respectively [165]. An inverse dose-response relation was noted for both PTB outcomes consistent with previous work linking maternal Vitamin D deficiency to bacterial vaginosis [166].

### 6.1. VDR Signaling in Preterm Birth

Experimental studies found associations between maternal Vitamin D status and placental antibacterial responses [30,33]. Toll-like receptors initiate the innate immune defense against microbial pathogens. Vitamin D reduces bacterial infections by inducing cathelicidin in decidua and cytotrophoblasts [139,167]. Low Vitamin D impairs toll-like receptor induction of the antimicrobial peptide cathelicidin from systemic macrophages [139]. Furthermore, calcitriol decreases expression of cytokines, such as granulocyte macrophage colony stimulating factor 2 (GMCSF-2), TNF-α, IL-6. LPS increases inflammatory reactions in myometrial tissue [168] and myometrial smooth muscle (UtSM) cells [169]. LPS and IL-1β trigger preterm delivery in rodent models [170,171]. Vitamin D decreases both LPS- and IL-1β-induced proteins in UtSM cells [172]. Therefore, Vitamin D attenuates the inflammation-induced expression of contractile-associated proteins in UtSM cells [34].

### 6.2. Role of VDR Polymorphisms in Preterm Birth

Information is sparse about association between preterm birth and *VDR* polymorphisms. *BsmI, TaqI, ApaI, FokI* SNPs were investigated by comparing maternal and neonatal genotype frequencies between the term and preterm cohorts.

In an Isreali population, the genotype frequencies of the *VDR ApaI* polymorphisms differed between term and preterm cohorts. Women carrying the *VDR ApaI* homozygote genotype had a higher risk for preterm births compared to the heterozygote group. This remained significant after adjustment for confounders. They couldn’t find a link between *VDR BsmI*, *TaqI* or *FokI* variation frequencies between preterm and term births [173].

Manzon et al. investigated *FokI, ApaI, TaqI* and *BsmI* SNPs concerning the risk of spontaneous preterm birth in an Israeli population. In this population, maternal *FokI* and *TaqI VDR* allele frequencies were significantly different in the preterm birth group. However, only maternal *FokI* variant could be linked to an increased risk of preterm birth (odds ratio OR = 3.317). The ORs for the other variants such as *TaqI, BsmI* or *ApaI* were insignificant [19]. In addition, work from a polish group indicates that individual maternal SNPs of the *VDR* gene, namely the *TaqI, BsmI* and *ApaI* polymorphisms, had no effect on preterm birth. But they investigated some genotype combinations and e.g., some combinations reduced the risk for preterm birth significantly [174]. Polymorphisms of *VDR*, which are associated with pregnancy complications, are listed in Table 1.

## 7. Existing Knowledge from VDR Knock-Out Models

As elucidated above, common pregnancy complications are associated with impaired trophoblast function and impaired immune regulation on the feto-maternal interface. Even if we have multiple experimental and observational studies, a clear mechanistic link between VDR expression and placental function and therefore fetal outcome is still missing. Proper designed investigations with VDR knockout dams may add important knowledge in this area:

VDR knockout (*Vdr^−/−^*) mice had lower fetal weights in comparison to heterozygous (*Vdr^+/−^*) animals [175]. As mentioned above, investigations in knockout VDR and Cyp27b1 knockout mice showed that Vitamin D controls placental inflammation [34]. Wilson et al. found no differences in morphology of *Vdr*^−/−^ fetus or placenta, when the *Vdr^+/−^* mother was fed with sufficient vitamin D and calcium [176]. Dams with a vitamin D deficient diet had reduced placental size [177]. The authors concluded that it might be the decidua, which mediates the impact of low maternal vitamin D on the placenta, rather than direct VDR signaling in the placenta [176]. Interestingly, the authors detected that especially some genes which are involved in oxidative stress and fetal growth are different between *Vdr*^−/−^ and *Vdr*^+/+^ placentae [176]. This might be a link to development of pregnancy complications like preeclampsia [178].

## 8. Conclusions

To conclude, the evidence out of experimental studies is convincing, Vitamin D together with the placental nuclear receptor VDR has important impact on the fetomaternal unit in multiple pathways. VDR influences trophoblast differentiation and function, regulates immune reactions and inflammation. However, existing clinical studies give conflicting results about the impact of vitamin D in pregnancy disorders. Vitamin D status is influenced by dietary habits, skin pigmentation, sun exposure, adiposity and missing standardization of measurements. These factors hinder proper design and reproducibility of clinical studies. Furthermore, the relationship between a polymorphism and phenotype cannot be clarified upon in studies where individual SNPs are analyzed in isolation and interpretation of association studies involving the *VDR* gene are difficult because most of the SNPs are non-coding. The aim of further designed studies should be to investigate the functional haplotype structure of *VDR* polymorphisms in different populations. 

Taken together the knowledge about vitamin D and placental VDR, we assume that disturbances in VDR expression and function, which result in pregnancy complications, may refer mainly to the maternal part of the placenta. Information about the consequences of defective VDR signaling in the fetus or the fetal part of the placenta are still missing, especially regarding the long time outcome of the offspring. Further investigations, which distinguish properly between fetal and maternal parts of the placenta and provide data about fetal programming, are warranted for the prevention of vitamin D related diseases in the offspring.

## Figures and Tables

**Table 1:** Vitamin D Receptor (*VDR*) polymorphisms in pregnancy complications.

Polymorphism	Pregnancy Complication
*FokI*	Preterm birth
	Gestational diabetes
	Influence on offspring size
*ApaI*	Preterm birth
	Gestational diabetes
	Influence on Birth weight
*TaqI*	Gestational diabetes

Table 1 shows the most common polymorphisms, which are associated with pregnancy complications in certain populations.

## Abbreviations

CAMP	Cathelicidin
EVT	Extra villous trophoblast
FGR	Fetal growth restriction
GDM	Gestational diabetes mellitus
LPS	Lipopolysaccharide
PTB	Preterm birth
SGA	Small for gestational age
T1DM	Type 1 diabetes mellitus
T2DM	Type 2 diabetes mellitus
TL4	Toll-like receptor 4
UtSM	Myometrial smooth muscle
VDR	Vitamin D receptor
VSMC	Smooth muscle cells of the placental vessels
